# Exploring healthcare provider and patient perspectives on current outpatient care of venous leg ulcers and potential interventions to improve their treatment: a mixed methods study in the ulcus cruris care project

**DOI:** 10.1186/s12875-022-01841-5

**Published:** 2022-09-08

**Authors:** Regina Poß-Doering, Carolin Anders, Thomas Fleischhauer, Joachim Szecsenyi, Jonas.D. Senft

**Affiliations:** 1 Dept. of General Practice and Health Services Research, University Hospital Heidelberg, Heidelberg, Germany; 2 Institute of Medical Informatics, University Hospital Heidelberg, Heidelberg, Germany

**Keywords:** Venous leg ulcer, Standard operating procedure, Disease management, Mixed methods study

## Abstract

**Background:**

The project "Ulcus Cruris Care" aims to improve primary care for patients with venous leg ulcer (VLU) in General Practitioner (GP) practices using a complex intervention comprised of educational components, standardized treatment recommendations, computer-assisted documentation, and case management by non-physician medical assistants (MAs). Prior to implementing and testing the intervention components in general practices, in-depth exploration of current outpatient treatment of VLU patients and relevant implementation determinants was pursued.

**Methods:**

A mixed-methods study explored views of GPs, MAs, and patients regarding current VLU outpatient care and the planned intervention components to identify potential implementation determinants. Data were collected through semi-structured guide-based telephone interviews (*n* = 29) and a survey questionnaire (*n* = 28). Interviews were transcribed verbatim. Analysis was inductive initially and finalized in a deductive-inductive approach based on domains of the Theoretical Domains Framework to support structuring of relevant implementation determinants. Survey data were analyzed descriptively.

**Results:**

Current VLU outpatient care was described as frequently tailored to individual wounds and gradient. In general, workload was shared by GPs (diagnostics, counselling) and MAs (wound care). All care providers were aware of compression therapy, yet not all of them considered it essential for VLU care. Standardized operating procedures and educational components including e-learning were considered supportive. Stronger involvement of non-physician assistants was seen as opportunity to optimize VLU care. Concerns were identified regarding integration of software-supported case management into daily practice routines and regarding potential limitations in decision-making autonomy when using standard operating procedures.

**Conclusions:**

Findings in this study emphasize a need for educational interventions addressing VLU care providers as well as patients, particularly with regards to compression therapy. The conception of the planned intervention appears to be adequate and a structured guideline-based case management might be a promising approach for optimization of VLU treatment.

## Background

In developed countries, venous leg ulcers (VLUs) have a prevalence of 1–2% of the population and account for approximately 70% of chronic leg ulcerations [1, 2]. Due to rising age and comorbidity a steadily growing prevalence can be expected [3]. With an estimated mean prevalence of up to 0.64, approximately up to 1.8 million people in Germany are affected [4].

VLU patients experience a severe reduction in quality of life, primarily due to pain, disability, and high recurrence rates [5–9] and wound care is associated with a high nursing and medical expenditure [5, 10, 11]. Wound healing might take months and impair affected patients in their daily activities and social participation, resulting in reduced quality of life and high prevalence of psychological disorders [1, 6, 12, 13]. An increased use of the healthcare system and subsequent costs are to be expected as well [14]. On the other hand, outpatient care of VLU patients appears to entail considerable deficits. Care analyses show that compression therapy, which is known to be highly efficient in promoting wound healing, is applied in merely 30–50% [15–17], though evidence shows effectiveness in preventing VLU recurrence [18]. Such under‐usage of compression therapy thus represents a serious issue for VLU management [19]. Studies also indicate insufficient knowledge about compression therapy and practical application among care providers [19–21] as well as unclear pathways for referral [19]. Furthermore, current wound management concepts focus strongly on local wound therapy and wound dressings and might promote a rather passive role in patients, which may be reflected by lacking patient information and adherence [19, 22]. As a consequence, management of VLU is often not performed according to best evidence and affected patients may suffer from prolonged wound healing duration and avoidable chronification, although patient preferences seem to be compatible to clinical practice guidelines [23].

Medical societies in Germany see an urgent need for a standardized VLU treatment strategy via a multi-professional and multi-disciplinary approach with GPs in a central role [24, 25]. The Ulcus Cruris Care (UCC) project therefore aims to support outpatient VLU care in general practices by developing, implementing and evaluating a standardized evidence-based care concept similar to known disease management programs [26]. Development of the multi-faceted intervention in UCC includes training for GPs and non-physician medical assistants (MAs), standardized treatment recommendations based on current scientific knowledge, e-learning and print-based information for patients, as well as a software tool to support wound documentation and case-management to be implemented into administrative practice systems. The concept will be implemented in a pilot study accompanied by a process evaluation, followed by a randomized controlled trial to assess potential outcomes. Main target of the UCC intervention is to facilitate case management for VLU patients exerted by MAs and to support patient education and active patient participation in the treatment process. Furthermore, the UCC project intends to contribute new information and evidence to existing VLU literature that already indicates success of a standardized VLU care concept used by healthcare professionals for instance in long-term care facilities (paper-based forms) and home care services (via telemedicine) [27]. The upcoming UCC trial is intended to evaluate the effectiveness of the “Ulcus Cruris Care” intervention for the treatment of patients with VLU compared to usual care in the setting of German primary care practices [28].

Prior to implementing the concept, a preliminary study was conducted to capture subjective views of GPs, their non-physician medical assistants (similar to medical assistants in USA [29]), and patients regarding acceptance and perceived applicability of the developed intervention components to facilitate target group-specific refinement and support successful implementation into daily VLU care. Hence, this preliminary study aimed to explore (1) characteristics of current VLU therapy in primary care from the perspective of GPs, MAs and patients, and (2) the target group perspectives on the developed components prior to their implementation in the UCC project.

## Methods

### Study design

This non-interventional cross-sectional study used a mix of qualitative and quantitative methods to explore GP, MA, and patient perspectives on currently followed approaches to VLU wound care and their perspectives on the intervention components to be implemented in the UCC project. Semi-structured, guide-based interviews were supplemented by a printed one-time study-specific survey questionnaire and complemented by a sociodemographic questionnaire.

### Implementation program

The UCC project intends to use a multifaceted intervention program to implement a disease management concept for outpatient treatment of VLU patients in GP practices and evaluate its’ effectiveness in a prospective cluster-randomized controlled multicenter trial [30]. The intervention comprises four major components: (1) Online training and e-learning courses for GPs and MAs as well as e-learning courses and print information for patient education; (2) Evidence-based Standard operating procedures (SOPs) for VLU treatment; (3) Software supported case management; (4) Strong involvement of practice team and patients.

### Study population

As per (un-published) study protocol and based on experiences in prior research projects, a sample size of 30 participants was aimed for (GPs *n* = 10, MAs *n* = 10, and patients *n* = 10). MA is used as an umbrella term for support staff in GP practices and includes staff with different qualifications [29]. Inclusion criteria asked for all participants to be at least 18 years of age, legally fully competent and in fluent command of German. Participating GPs had to be practicing in the German federal state of Baden-Wuerttemberg, MAs had to be involved regularly in wound care in the practice and patients had to be in treatment for a VLU either during or within the last 12 months prior to the study period. Patients with arterial or mixed arterial VLUs were excluded. Participants who were enrolled for the UCC intervention were excluded from participation in the preliminary study to avoid contamination bias.

### Recruitment and sampling

Invitations to participate in an interview and the study-specific survey were extended to practicing GPs, MAs working in GP practices and routinely involved in chronic wound treatment, as well as patients who had been seeking treatment for VLU at a GP practice or at the wound outpatient clinic of the University Hospital Heidelberg. A convenience sampling strategy was followed. GPs and MAs personally known to the project management from prior research activities and from academic teaching practices were contacted via phone and invited for participation. Patients were recruited by purposive sampling during consultation hours in the wound outpatient clinic of the University Hospital Heidelberg (direct approach of suitable patients by a study team member), and in academic teaching practices. Participating practices could support and initiate patient recruitment by addressing eligible patients.

All interested parties meeting the inclusion criteria received written and verbal information regarding content and aim of the study and respective data protection regulations. A signed letter of intent had to be returned to be included in this study. Sufficient time was provided to give written informed consent for participation prior to data collection. In addition to participating in an interview and the study-specific survey, recruits were asked to fill in a one-time socio-demographic questionnaire. No reimbursement was provided. The targeted sample size was set at *n* = 30 (GPs *n* = 10, MAs *n* = 10, patients *n* = 10).

### Data collection

Semi-structured guide-based telephone interviews were conducted by three members of the study team (CA, TF, RPD). All interviewers had a background in health sciences and health services research and were experienced interviewers. Participants were interviewed at home or at their workplace. Interviewers conducted the interviews either from their workplace or from their home office. Based on the pre-defined research questions, the interprofessional team (Health Services Research, General Practice) of researchers (CA, RPD, TF, JDS) developed study-specific interview guides for the three groups of interviewees in an iterative process of collecting, discussing and subsuming appropriate questions and wording [31]. The interview guides were divided into two thematic sections to explore (1) current outpatient VLU wound care approaches in primary care and related experiences and (2) perspectives on the planned intervention components. Separate interview guides were developed for each target group. The first interview in each group served as a pilot and minor wording adjustments were included afterwards. After the first five interviews in each group, wording in interview guides was slightly adapted again where considered appropriate. All interviews were digitally recorded and transcribed verbatim. All data collected in interviews, survey and socio-demographic questionnaires were pseudonymized, electronically saved and stored on secure servers at the Department of General Practice and Health Services Research, University Hospital, Heidelberg.

A study-specific, non-validated questionnaire was developed to elicit perspectives on the planned intervention components and their perceived relevance. The questionnaire comprised five main items (1) standard operating procedures; (2) educational components for GPs, MAs and patients; (3) software-supported case management; (4) involvement of non-physician practice assistants; (5) classification of therapy and educational elements and corresponding sub-items to be scored in a 5-point Likert scale (very high approval, high approval, partial approval, high disapproval, very high disapproval) and two free-text questions on outpatient VLU care. Socio-demographic characteristics of interview participants were collected with a separate questionnaire. All survey instruments were piloted for comprehensibility and practicability with the first participants of each target group and subsequently wording was slightly adapted.

### Data analysis

For the qualitative data, a two-phased thematically structured analysis was performed (inductive-deductive). In an inductive framework analysis [32], interview transcripts and free-text answers from questionnaires were organized, managed and analyzed in MAXQDA 2020 (Release 20.2.2). Framework analysis is a systematic matrix-based data structuring approach which is independent from epistemological basic assumptions and research styles and enables cross-case and cross-category comparison of qualitative data to identify central statements and potential contrasts [33]. In this initial step, inductive categories were formed referring to current processes in VLU wound care as reported by the first 5 participants in each group. Categories were confirmed when applied to the data generated from participants 5–10 in each group.

Based on the assumption that implementation of new intervention components requires behavioral adaptation among the individuals involved, the Theoretical Domains Framework (TDF) served as analytical framework in the deductive-inductive second analysis phase of the qualitative data [34, 35]. To represent the complexity of behavioral and organizational influencing factors, the TDF consolidates 33 behavioral theories and models and maps them into a framework of 14 theoretical domains. Application of the TDF provides a basis for adaptation of considered intervention components according to identified needs, barriers and facilitators [34]. Data were first deductively categorized by assigning themes to appropriate domains of the TDF and inductively complemented by themes emerging directly from the data.

Descriptive analysis of sociodemographic and study-specific questionnaires was performed using Microsoft Excel software (Versions 2101 and 1808). Means, standard deviations (SD), medians (MED), maximum and minimum values, and absolute and relative frequencies were calculated. To account for items with a high range between extreme values, percentiles and interquartile ranges were calculated as well. For the study-specific questionnaires, the verbal Likert scale was numerically recoded from 1 to 5 (disagree = 1, somewhat disagree = 2, partially agree = 3, somewhat agree = 4, agree = 5). Individual questionnaire items depicting the same thematic construct were combined into core statements and served to support interview statements.

Analysis for all data collected from participants 1–5 in each group first was performed by a junior researcher in the study team (CA) and reflected in methods workshops with junior and senior researchers. Subsequently, overall analysis of all data collected in the preliminary study was performed by experienced members of the study team (TF, RPD). Codings of the qualitative data were discussed and in case of divergence approximated to achieve a high intercoder congruence.

## Results

**Fig. 1:**
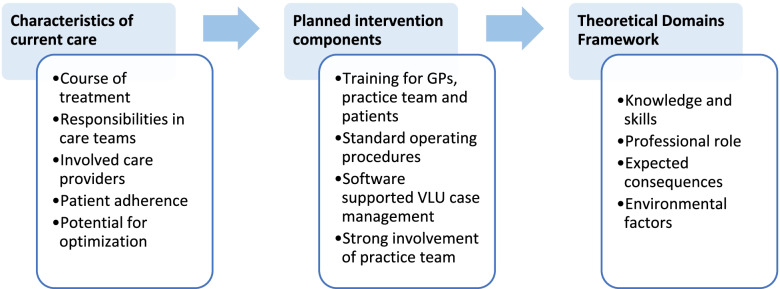
Theorizing analytical approach. Illustrates the methodical approach in this study. Relevant contextual factors describing the current course of VLU care were identified from survey and qualitative data. Data referring to the planned intervention components were categorized according to selected domains of the Theoretical Domains Framework where applicable to facilitate an explanatory framework for findings

Findings deriving from both, qualitative and quantitative data, are first presented with a focus on the description of current course of VLU treatment in GP practices and associated responsibilities and perceptions. Regarding perceptions of the planned intervention components in the UCC project, findings are categorized based on selected domains derived from the TDF which relate not only to relevant contextual factors expected to be identified from the quantitative data, but also to participant perceptions with regards to knowledge and skills, professional roles and beliefs about potential consequences of the planned innovation as derived from the qualitative data. The theorizing analytical approach is described in Fig. 1.

### Participant characteristics

Between August 2020 and March 2021, a total of 31 interviews were conducted: GPs (*n* = 10), MAs (*n* = 11), and Patients (*n* = 10). One patient interview and corresponding survey questionnaire could not be included for analysis as consent was withdrawn after the interview. To avoid potential data duplication and contamination bias, one MA interview and questionnaire were not considered in the analysis since the MA was included in the UCC study intervention group past the interview. Due to poor quality of the recording, one patient interview was transcribed only partially. One MA survey questionnaire was irretrievably lost in the mail and the MA did not provide a replacement. Table 1 describes characteristics of all participants included in this study (*n* = 29).

**Table 1:** Participant characteristics (*n* = 29)

	**GPs** (*n* = 10)	**MAs** (*n* = 10)	**Patients** (*n* = 9)
**Age** mean (SD), range	50 (10.8), 34-62	46 (13.1), 28-62	69 (10.5), 51-80
**Gender** n (%) male	8 (80 %)	0 (0 %)	4 (44%)
**Medical specialty** - n (%)			
General Practitioner	5 (50 %)		
Internal Medicine	5 (50 %)		
**Completed specialization training**^a^ n (%)			
Non-physician practice assistant^b^		8 (80 %)	
Case Management		2 (20 %)	
Wound care		2 (40 %)	
**Experience in years **mean (SD), range	21 (9,9), 7-33	25 (12.2), 6-41	
**VLU present since number of months **median (SD), range			17.9 (145.2),5-429^+^
**VLU-related visits to GP per month** mean (SD), range			5.6 (3.7), 1-11

^a^ multiple answers were possible

^b^ qualification exceeding general vocational training to perform delegated tasks achieved in programs with different scopes (such as home visits)

^+^ includes two patients with recurring wounds and referring answers

### Characteristics of current outpatient VLU care

#### Treatment, responsibilities and involved care providers

All GPs reported to be responsible for diagnosing patients with VLU, determining therapy, providing patient counselling and education, and coordination of interdisciplinary clarification. MAs were included in patient counseling and wound care. Modern moist wound therapy, compression therapy, exercise and elevation of the leg were the main VLU therapy recommendations mentioned. GPs (*n* = 7) and MAs (*n* = 5) emphasized the importance of compression therapy considering it more essential than local wound therapy. GPs stated that in some cases diagnostic clarification by phlebologists, angiologists, or surgeons was necessary. Outpatient nursing care, family members, dermatologists, wound centers, and—in rare cases -psychological care were identified as further potentially involved care providers.

Wound dressing changes in the GP practices were often performed jointly by GPs and MAs. GPs mentioned also to delegate compression therapy to MAs. MAs specifically qualified for delegated tasks in general practice and/or as wound assistants were tasked with GP-directed wound management as they were considered to have the appropriate level of training and expertise. Therapy was reported to be often adapted individually because of large differences between cases. Necessary extensive treatment efforts and high associated costs were discussed. In the survey data, a mismatch was observed regarding responsibilities in practices. While patients and MAs indicated that the trained physicians should mainly be responsible for local wound therapy and patient counselling (MED 3 = partial approval), GPs themselves disapproved (MED 2 = high disapproval) indicating their approval to delegating specific tasks.

The interviewed GPs only briefly talked about providing educational information to VLU patients. One GP perceived that time for patient education was limited, another GP stated to leave this to the MAs and often ‘leave the room [during patient education], since I am very confident in my well-trained MAs.’ (GP01, #35). Regarding educational information refering to their VLU, most patients stated they had received some information related to the relevance of compression therapy, exercise and regular changing of wound dressings by MAs rather than their GPs. One patient explicitly stated that more information and counselling would have been appreciated. Another patient particularly emphasized the relevance of a professional nursing care service regarding the competent change of the wound dressing and noted that the GP ‘opened this door for me’ (Patient08, partial transcript). The study-specific survey questionnaire included a self-reported assessment referring to relevance of frequently used therapy elements in VLU treatment. Table 2 describes the respective results.

**Table 2:** Assessment of relevance of therapy elements in VLU treatment

Elements	GPs	MAs Median	Patients
Wound treatment according to respective phase	4.5	5	5
Wound dressing type	4	5	5
Promote mobilization	4	5	5
Medical compression stocking	5	5	5
Medical compression dressing	5	5	4
Medical adaptive compression systems	3	4	5

5  very high approval, 4 high approval, 3 partial approval, 2 high disapproval, 1 very high disapproval

#### Patient adherence

GPs and MAs mentioned that VLU patients often were older persons and sometimes would not follow their advice well enough and consequently, wound healing often was slower than expected. GPs and MAs were aware of patients who administered self-chosen treatment options such as using ‘aloe vera leaves or honey ’ (GP09, #90) on their VLU. GPs also reported encountering difficulties regarding patient tolerance and adherence to compression therapy.

‘[…] we cannot repeat it often enough, how important compression is, […], but the question is really how we can increase acceptance there, because of course I can understand that when there is a 35 degree heat out there, one is not so willing to wear an elastic compression dressing.’ (GP02, #42)

‘[…] often we see patients who poorly or reluctantly want to accept compression therapy.’ (GP08, #20)

#### Potential for optimization

GPs expressed their intention to train more MAs in wound care to facilitate their broader involvement in wound management. Possible optimization was also noted for patient education and follow-up care with regular appointments to avoid recurrences. One GP explained that one ‘somehow would like to see basic wound care training offered to practicing physicians’ (GP01, #15) to optimize own competencies. GPs, MAs as well as patients saw potential for improvement of current outpatient VLU care in the area of prevention with a focus on early diagnosis and compression therapy. Referring to interprofessional care, aspects of communication with outpatient nursing services and their qualifications regarding VLU care were mentioned. One patient perceived GPs to be sometimes overwhelmed with the treatment of VLU which was partially confirmed by a GP. A lack of experience, expertise and training in dealing with chronic wounds, and a seemingly low number of VLU patients in the practice were seen as reasons. In addition, one patient perceived that in most GP practices MAs were not qualified for wound management. It was also mentioned that pain management needed more consideration.

‘[…] it is a very painful thing and I was not told that I could take regular pain medication. I always thought it was a god given and I had to endure the pain.’ (Patient06, #38)

### Perceptions regarding potential intervention components

Participant perceptions regarding the intervention components to be offered in the UCC intervention are reported based on the selected domains derived from the TDF.

#### Knowledge and skills

The survey participants were asked to indicate their importance classification regarding potential therapy elements for VLU outpatient care. Classification of listed elements was homogeneous and all three groups considered them to be very important or important. Exception here was the GPs’ only partial approval for patient instruction on best practice professional and unassisted wound dressing change. Table 3 shows the median classification.

**Table 3:** Importance classification of specific therapy elements for VLU outpatient care

Elements	GPs	MAs Median	Patients
Informing patients about the cause of disease	5	5	4.5
Educating patients about preventive behavior	5	5	5
Promote mobilization	4	5	5
Instruct patients on proper application of compression dressing	4.5	5	4
Instruct relatives on proper application of compression dressing	5	5	5
Instruct patients on correct unassisted dressing change	3	5	4
Instruct relatives on correct unassisted dressing change	4	5	5
Guiding patients to increase mobility	4.5	5	4
Strong involvement of non-physician assistants in VLU case management	4.5	4	5

5 very high approval, 4 high approval, 3 partial approval, 2 high disapproval, 1 very high disapproval

#### Educational components

In the interviews, educational components for GPs and their assistants were mostly viewed positively and considered important. A need for training was seen justified by existing deficits related to procedural knowledge regarding the description of the wound status, dressing materials and changes, compression therapy, updating of professional knowledge against the background of new medical developments and therapeutic standards. In contrast to the care provider’s perceptions who mentioned patient age, cognitive impairment, potentially low digital affinity and availability of digital devices as restricting factors, all patients expressed interest in participating in training, for example regarding pathogenesis of wounds and compression therapy. They were supportive of an offer of e-learning-based training as well as written, paper-based information material. E-learning formats were generally seen as fitting and with increasing acceptance, particularly during the COVID-19 pandemic. On-site training was mentioned to have the clear advantage of opportunity to ask questions and feedback. It was suggested to also include outpatient care services in training activities. There was consensus that compression therapy and how to properly apply it should be covered by training offers also for nursing care staff.

‘An incorrect wound compression by nursing care staff caused a decubitus ulcer that got infected, […] and then I needed an operation […]. So, I think [educational] interventions targeting the professionals are more important than patient education.’ (Patient08, partial transcript)

Regarding the potential content of educational intervention components for patients, a very high approval from GPs, MAs, and patients was observed in the survey data for all explored items (MED 5 = very high approval). Findings confirmed the understanding gained from the interview data regarding e-learning and educational material. Again, there was a mismatch observed between care provider and patient views, particularly regarding the patient view on training activities involving their relatives where they only partially approved (MED 3) while providers stated a very high approval. A further mismatch was observed for e-learning which found high approval by patients (MED 4), yet only partial approval by the health care professionals (MED 3.5 and 3). Table 4 details survey findings regarding potential training content and views on e-learning and educational material.

**Table 4:** Views regarding contents and form of potential training material for patients

	GPs (*n*=10)	MAs (*n*=9)	Patients (*n*=9)
		Median	
Cause of disease	5	5	5
Preventive measures to avoid recurrences	5	5	5
Professional compression therapy	5	5	5
Professional local wound care	4	5	5
Mobilization and exercise	5	5	5
Awareness for complications	5	5	5
Patients appreciate written informational material	4	5	5
Patients would welcome e-learning based training	3	3	4
Patients benefit from written educational material	4	5	4
Patients benefit from e-learning based training	3,5	3	4
E-learning should also address patients’ relatives	4,5	5	3

5 very high approval, 4 high approval, 3 partial approval, 2 high disapproval, 1 very high disapproval

#### Standard operating procedures

During the interviews, participants in all three groups indicated a generally positive view on implementing a guideline-based approach to VLU outpatient care. Patient perception indicated that ‘This is useful in any case’ (Patient07, #175). GPs and MAs reported positive experiences with disease management programs and existing quality management standards and based their positive outlook on these prior experiences. Potential restriction of GPs’ freedom of choice of therapy when following a guideline-based approach were contemplated as ‘… own experience as a GP must be allowed for consideration as well.‘ (GP03, #36).

‘ … I think through the standardized disease management program approaches to diabetes care etc., we improved therapy, so overall, standardization would not be bad …’ (GP03, #32)

In the survey, GPs, MAs and patients also indicated a high approval for the introduction of standard operating procedures for VLU care (MED 4 = high approval). They stated that guidance by standard operating procedures could improve the quality of VLU outpatient care (MED 5 = very high approval). All three participant groups indicated that such standards might limit the GPs choices for individual therapy options (MED 3 = partial approval).

### Professional role

#### Strong involvement of practice team

During the interviews, a more central role of MAs in VLU outpatient care was viewed positively in all three participant groups regarding wound care, compression therapy, and patient education. Sharing responsibilities during the care process was seen as a potential motivational incentive for MAs by GPs, patients, as well as MAs themselves. GPs emphasized they would like to hand specific delegable tasks to qualified non-physician practice assistants and perceived this ‘would increase the significance of their contribution to care processes ‘ (GP08, #42). They also expected to achieve a workload reduction in doing so. Sharing care responsibilities was also seen as contribution to optimized care quality as it was expected to improve therapy adherence.

‘Yes, I think this is great, because they might find a better connection to the patient than us, and when patients hear information repeatedly, it might reach them a little better. So, the more times they hear ‘you have to exercise more often, you need to compress consequently’, I think this is great, because constant dripping wears the stone.’ (GP07, #50)

The survey data showed that GPs disapproved the idea of local wound therapy, patient guidance and counselling being their professional domains exclusively (MED GP 2 = high disapproval) while MAs and patients gave a more cautious assessment (MED MAs and patients 3 = partial approval). A potential contribution to optimizing care quality for VLU patients was also seen in using software-supported VLU case management (MED approval GPs = 4, approval MAs = 4.5).

#### Expected consequences

Patients perceived the benefit of training for physicians to be in an increased awareness of prevention and improved knowledge about chronic wounds such as VLU. The benefits of patient education were seen in improved self-monitoring and prevention of recurrence, more active patient involvement in the treatment process, and promotion of treatment adherence, particularly with regard to compression therapy.

‘I also think it [training] would be helpful, because many patients find leg wrapping or compression, meaning compression therapy as a whole, to some extent annoying and don't want to do it.’ (GP04, #47)

GPs expected that guideline-based VLU care could facilitate a more uniform treatment approach and provide confidence that ‘you then just know, okay I'm on the safe side if I do it this way’ (GP06, #62).

One GP also mentioned that using a guideline-based approach would enable ‘critical reflection’ (GP GP09, #236) of own routines. MAs considered working along a guideline to be a ‘probably good decision support for the GP’ (MA07, #124), to support particularly less experienced physicians and to bring everybody involved ‘on the same level’ (MA09, #154). One patient expected that ‘First of all, wound healing will improve’ (Patient07, #129). The survey data showed that a guideline-based approach to VLU care was considered to facilitate informed therapy decisions and provide more time for care. All three participant groups saw potential for a high added value with respect to optimization of care quality (MED 5 = very high approval).

### Environmental factors

#### Software supported VLU disease management

Regarding a software supported VLU case management, GPs and MAs saw advantages in potentially reduced workload and time needed as well as fast access to standardized documentation. All three participant groups considered that software supported case management could add more transparency throughout the course of treatment. The care providers stated that a software supported VLU case management should not be cumbersome and extensive. They emphasized that sufficient interoperability and compatibility with existing administrative systems had to be given to avoid isolated solutions, time expenditure and unnecessary duplication of documentation. One of the GPs and one patient mentioned concerns referring to privacy of sensitive patient data.

‘ […], then you would have it [the documentation] in a standardized format, and you could look at nice trajectories […].’ (GP09, #232)

‘It is probably important that there is added value for MAs, GPs, and patients, because the question is also whether we can support the patient with parts of the documentation […].’ (GP10, #52)

’[…] wound check could take place in more regular intervals than before perhaps, you might plan appointments and the patient cancels, and then it is forgotten, but then with the software, you might get a reminder, since often something pops up, right. (MA06, #148)

## Discussion

This study aimed to explore current VLU outpatient care in general practices in Germany and perceptions regarding intervention components planned to be implemented during the intervention period of the UCC project. In Germany, the majority of outpatient VLU care is provided by GPs [22] which highlights the centrality and significance of this setting. Participants described the current division of tasks between GPs and MAs and the network of a number of care providers who potentially might be involved in VLU outpatient care. Regarding the planned intervention components, participant perceptions of educational components for GPs, MAs and patients were generally approving, yet contrasted regarding adequate formats. Standard operating procedures were seen as adequate guidance for MAs and particularly for less experienced GPs, but there were concerns about possible limitations for individually tailored therapy options. Provided a sufficient interoperability and compatibility with existing administrative systems, software supported case management was considered beneficial. A stronger involvement of adequately trained non-physician assistants in VLU outpatient care was viewed positively by GPs, yet patients and MAs only partially approved of this component.

Caregivers and patients in this study both were aware of knowledge deficits in all parties involved in VLU outpatient care regarding wound care, compression therapy and therapeutic standards. This was particularly contemplated regarding the involvement of outpatient nursing care and family members, emphasizing the need to include them in educational activities aiming to provide literacy about VLU care and facilitate active patient participation in related processes. Studies in the field reported that such active patient participation may help to improve VLU outcomes and can be supported by evidence-based patient education about the significance of compression and exercise [19, 22]. Consequently, the UCC intervention aims to actively integrate patients into the treatment process by using such evidence-based standardized educational efforts to shift the focus from expert wound dressing changes to a more interpersonal approach in VLU treatment.

Prior studies indicated insufficient knowledge about compression therapy and its’ practical application among care providers [20, 21]. In this study, participants had some knowledge about compression therapy, yet not all care providers considered it as a central element of VLU outpatient care. Evidence-based compression therapy is known to be highly efficient in VLU treatment [16] and can reduce healing time and risk of recurrence [5, 36]. However, analyses in Europe showed that it is received by around 40% of VLU patients only and device and modalities of compression are often chosen inadequately [22, 37]. In Germany, high-quality clinical guidelines referring to VLU treatment are in place [25, 36, 38], yet this rate is very similar, suggesting insufficient implementation in daily care processes [39]. A strong caregiver-sided focus on local wound therapy and a lack of patient knowledge about the benefits of compression therapy may contribute to a rather passive patient role and adherence deficits as described by GPs in this study. Furthermore, reports in this study on patients’ self-chosen treatment options such as applying honey or aloe vera leaves indicate that patient-sided misinformation is prevalent. This emphasizes the need to regularly address and integrate patient education as a central pillar in the VLU care process. At the same time, the perceived insecurities and lack of expertise regarding compression therapy as well as the fact that only two participating MAs had specific wound care training point to a need for educational efforts on provider side as well. Since a more adequate application of compression therapy and devices presumably has a joint positive impact on therapy adherence, wound healing and treatment duration, it can be assumed that educational efforts for care providers as well as patients would be beneficial.

Participants in this study had a positive view on the UCC concept and referenced prior experiences with disease management programs to support this view. Such programs are well established in German primary care and are seen as contribution to structured patient care, therapy adherence, and expanded diagnostic and therapeutic knowledge [40, 41]. However, there were concerns mentioned particularly with regards to the amount of documentation required in a structured VLU care program and to a potentially reduced flexibility for tailoring treatment individually to wounds and patients. Presumably stemming from patient safety considerations, GPs also were concerned about a more autonomous change of wound dressings by patients, while the more approving rating by patients indicates appreciation for less expenditure on their side. All three participant groups shared the perception that educational components should cover key topics of VLU outpatient care, while more general aspects referring to nutrition or exercise were not contemplated extensively. This indicates an opportunity to adapt all intended training material to promote counselling accordingly to close this gap. With regards to the involvement of MAs in the care process, all GPs supported delegating tasks of patient guidance, counselling and wound care while MAs and patients were more hesitant and only partially approved. This indicates an even stronger need for adequate training and qualification to unfold the full potential inherent to task delegation in VLU outpatient care in general practice.

### Strengths and limitations

The presented study highlights characteristics of current VLU outpatient care. With the TDF, a methodologically adequate tool was applied to identify potential determinants for implementation of specific components in the complex UCC intervention. The mixed-methods approach provided added value for gaining insights into perspectives on planned components. The chosen multi-perspective approach which included physicians, MAs and patients strengthened findings particularly with regards to interdisciplinary challenges of VLU outpatient care. Reporting of this study was guided by the Consolidated Criteria for Reporting qualitative Research (COREQ) [42]. The study comprised a survey with 29 participants which might be considered as a small sample size. Nevertheless, the density of the qualitative data facilitated a thorough analysis and sufficient illustration of identified categories pointing to thematic saturation and an effective sample size as indicated by empirical guidance [43]. The analytical approach enabled identification of relevant themes and the achieved high inter-coder congruence reflects a reliable classification and robust understanding of the generated data and the study phenomenon. Qualitative and quantitative data were first analyzed separately and then combined to strengthen insights.

Some limitations have to be reported. Recruitment of interview partners via personal contacts and the direct approach of patients might have influenced assessments regarding the planned intervention components and potentially allowed for social desirability of answers resulting in an overestimation of acceptance. Also, patients might have had limited knowledge of medical terms used in this study which could have led to misinterpretations. The TDF was used during data analysis, not during design of the study or development of data collection instruments which could have provided a stronger methodological consistency.

## Conclusions

GP, MA and patient perspectives on current VLU outpatient care confirm a need for educational interventions addressing care providers as well as patients in order to improve VLU treatment, particularly, by promoting adequate use of evidence-based compression therapy. The findings of this study confirm adequate selection of intervention components in the overall Ulcus Cruris Care project and indicate that a structured, guideline-based case management approach might be key for VLU outpatient care optimization.

## Data Availability

All data generated and analyzed in this study are stored on a secure server at the University Hospital Heidelberg, Germany, Department of General Practice and Health Services Research. Due to data protection and privacy regulations, data is not available publicly. De-identified sets of the data collected and analyzed during this study can be made available by the corresponding author on reasonable request.

## Abbreviations

COREQ: Consolidated criteria for reporting qualitative research
GP: General practitioner
MA: Non-physician medical assistant
MED: Median
SD: Standard deviation
TDF: Theoretical Domains Framework
UCC: Ulcus Cruris Care
VLU: Venous leg ulcer
